# LncRNA NUTM2A-AS1 silencing inhibits glioma via miR-376a-3p/YAP1 axis

**DOI:** 10.1186/s13008-024-00122-0

**Published:** 2024-05-10

**Authors:** Yuecheng Zeng, Zhenyu Yang, Yang Yang, Peng Wang

**Affiliations:** Department of Neurosurgery, Xiangyang Central Hospital, Affiliated Hospital of Hubei University of Arts and Science, No. 136 Jingzhou Street, Xiangcheng District, Xiangyang, 441021 China

**Keywords:** LncRNA NUTM2A-AS1, miR-376a-3p, YAP1, Glioma, Proliferation, Apoptosis

## Abstract

The lncRNA NUTM2A-AS1 has been shown to be dysregulated in gastric cancer, while the roles in glioma is unclear. The aim of this study was to investigate the roles and potential mechanisms of lncRNA NUTM2A-AS1 in the proliferation and apoptosis of glioma cells. The StarBase software and dual luciferase reporter assay were used to identify the relationship between lncRNA NUTM2A-AS1 and miR-376a-3p, and miR-376a-3p and YAP1. The expression of lncRNA NUTM2A-AS1, miR-376a-3p, and YAP1 in human glioma cell lines was detected by qRT-PCR. MTT and flow cytometry were used to detect the effects of lncRNA NUTM2A-AS1 or miR-376a-3p on the proliferation and apoptosis of U251 and A172 cells, respectively. In addition, changes of Bax and Bcl-2 expression in glioma cells were further verified by western blotting and qRT-PCR. The results showed that the expression of lncRNA NUTM2A-AS1 was elevated in glioma cell lines, while miR-376a-3p was decreased. LncRNA NUTM2A-AS1 was negatively correlated with miR-376a-3p. Silencing of lncRNA NUTM2A-AS1 enhanced the levels of miR-376a-3p, leading to reduced cell proliferation and increased apoptosis in glioma cells. YAP1 was a direct target of miR-376a-3p, and it was negatively regulated by miR-376a-3p in U251 and A172 cells. Further mechanistic studies suggested that miR-376a-3p reduced glioma cell proliferation and increased apoptosis by inhibiting YAP1 expression. In addition, lncRNA NUTM2A-AS1 positively regulated of YAP1 expression in glioma cells. In conclusion, silencing of lncRNA NUTM2A-AS1 inhibited proliferation and induced apoptosis in human glioma cells via the miR-376a-3p/YAP1 axis.

**Supplementary Information:**

The online version contains supplementary material available at 10.1186/s13008-024-00122-0.

## Introduction

Gliomas originate from glial cells in brain, and are one of the most common malignant tumors in central nervous system, with a high morbidity (30%) and mortality rate (3.3%) [1, 2]. Depending on the degree of malignancy, gliomas are graded from I to IV. Glioblastoma is grade IV and is the most aggressive malignancy [3]. Gliomas are currently treated clinically through surgery, radiotherapy and chemotherapy, but patients have a poor prognosis and short survival rate, eventually progressing to high-grade gliomas (grade III or IV) [4, 5]. However, the detailed pathogenesis of gliomas and effective treatments remain uncertain. Therefore, it has become necessary to study the pathological mechanisms of glioma at the genetic and molecular levels, which will facilitate the identification of new diagnostic and targeted therapeutic strategies.

Long non-coding RNAs (lncRNAs) are greater than 200 nucleotides in length and do not encode proteins [6]. Studies have shown that lncRNAs could achieve their biological functions by regulating gene transcription, acting as signaling molecules, scaffolding protein complexes, and participating in regulating a variety of biological processes [7, 8]. LncRNAs are widely present in tumors and their expression levels could influence tumourigenesis [9]. Several studies in recent years have revealed that lncRNAs are involved in regulating the progression of gliomas [2, 10]. For example, lncRNA LINC00319 regulates glioma development through directly binding to TATA-box binding protein-associated factor 1 (TAF1) [11]. In addition, lncRNA NEF could inhibit glioma progression by downregulating TGF-β1 expression [12].

Mechanistic studies have shown that lncRNAs act as sponges for competing endogenous RNAs (ceRNAs), regulating the function of microRNAs (miRNAs), thus forming a complex regulatory network involved in tumour development [9, 13, 14]. It has been reported that lncRNA FOXD2-AS1 regulates glioma progression through the miR-31/CDK1 axis [15]. Furthermore, lncRNA HCG11 could regulate glioma progression by sponging miR-496 and upregulating CPEB3 expression [16]. Recently, it found that LINC00473 directly binds to miR-195 as a ceRNA in glioma cells and participates in post-transcriptional signaling regulation [17, 18]. However, there are still a considerable number of lncRNAs whose function in gliomas are still unclear. Recent studies have shown that NUT family member 2A antisense RNA 1 (lncRNA NUTM2A-AS1), located on Chromosome 10, is upregulated in various types of cancer including non-small lung cancer, gastric cancer, hepatocellular carcinoma, and prostate cancer [19–22]. Silencing of lncRNA NUTM2A-AS1 has been reported to regulate the viability and apoptosis of lung adenocarcinoma cells (LUAD) by regulating the miR-590-5p/METTL3 axis [23]. However, the expression and roles of lncRNA NUTM2A-AS1in glioma remain unclear.

MiRNAs are a class of non-coding RNAs encoded by endogenous genes and are approximately 22 nucleotides in length [24]. MiRNAs are widely expressed in eukaryotic organisms and are involved in tumourigenesis [25, 26]. It has been reported that miRNAs can be used as potential biomarkers for the diagnosis of gliomas [27]. It was found that miR-376a-3p was lowly expressed in human glioma tissues, and upregulation of miR-376a-3p inhibited the aggressiveness of tumor and affected glioma development [28]. In addition, miR-376a-3p is associated with lymphatic metastasis in gliomas and attenuates glioma metastasis by negatively regulating KLF15 expression [29]. These findings suggest that miR-376a-3p plays an important role in glioma. It has been reported that lncRNA NUTM2A-AS1 sponges to miR-376a and is involved in gastric carcinogenesis [20]. However, the relationship between lncRNA NUTM2A-AS1 and miR-376a-3p in glioma is unclear.

Bioinformatics studies revealed that YAP1 was a potential target gene for miR-376a-3p. YAP1 is highly expressed in human glioma, and it may serve as a reliable prognostic biomarker and therapeutic target for glioma [30, 31]. Therefore, we speculate that the lncRNA NUTM2A-AS1 may regulate the malignant biological behavior of glioma cells by regulating the miR-376a-3p/YAP1 axis. The aim of this study is to explore the role of lncRNA NUTM2A-AS1 in glioma cells and analyze the underlying molecular mechanisms.

## Results

### LncRNA NUTM2A-AS1 sponges to miR-376a-3p

**Fig. 1:**
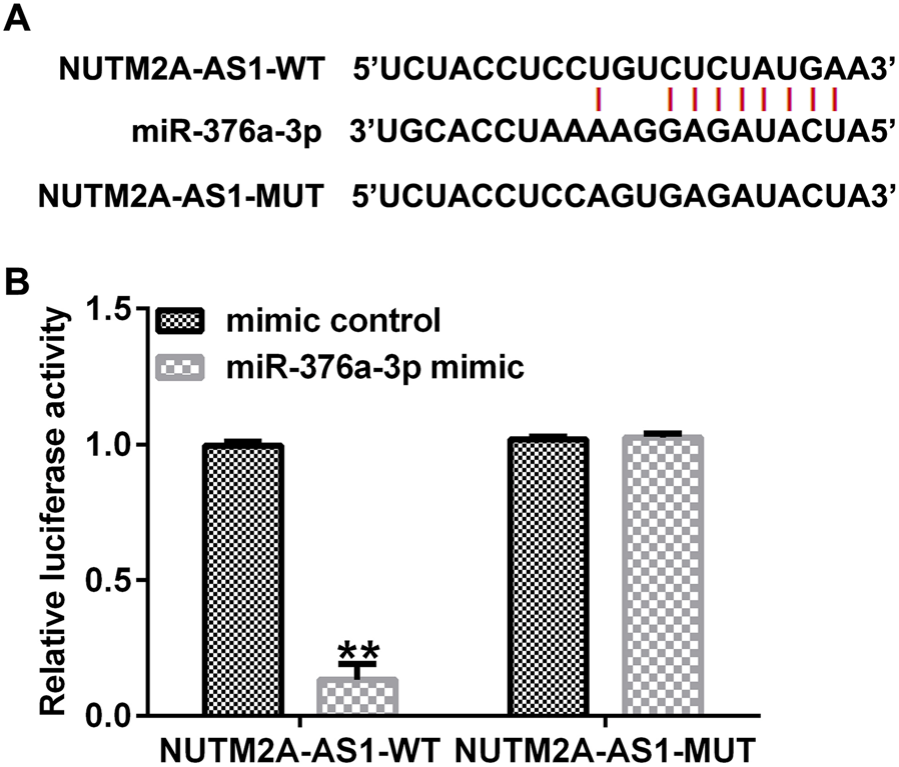
MiR-376a-3p is a direct target of lncRNA NUTM2A-AS1. **A** Binding sites between miR-376a-3p and lncRNA NUTM2A-AS1 were predicted with StarBase. **B** Dual luciferase reporter assay was performed to confirm the interaction between lncRNA NUTM2A-AS1 and miR-376a-3p. **p < 0.01 vs. mimic control group

To understand the mechanism of lncRNA NUTM2A-AS1 in glioma, we used the StarBase database to predict the potential targets of lncRNA NUTM2A-AS1 in glioma. The results showed the potential binding sites between miR-376a-3p and lncRNA NUTM2A-AS1 (Fig. 1A). Subsequently, the interaction between lncRNA NUTM2A-AS1 and miR-376a-3p was verified by a dual luciferase reporter assay. The results revealed that the luciferase activity of NUTM2A-AS1-WT was significantly lower than mimic control group after co-transfection with miR-376a-3p mimic (Fig. 1B). These results suggest that lncRNA NUTM2A-AS1 sponges to miR-376a-3p in glioma.

### Expression levels of lncRNA NUTM2A-AS1 and miR-376a-3p in glioma cell lines

**Fig. 2:**
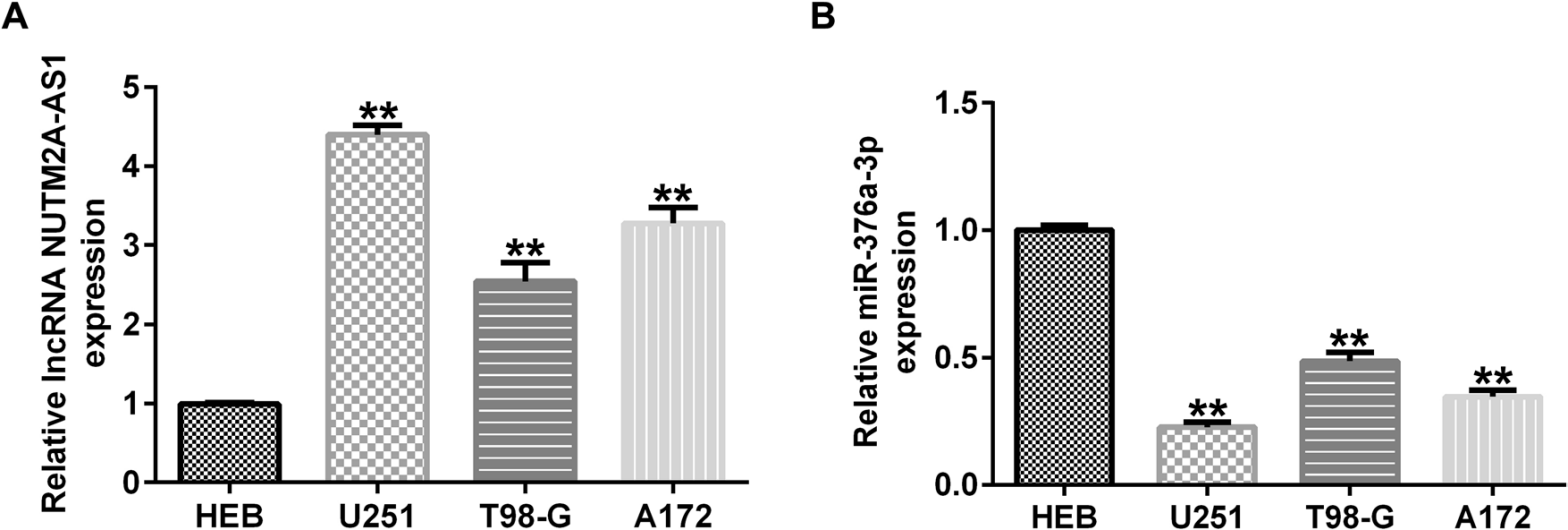
Expression levels of lncRNA NUTM2A-AS1 and miR-376a-3p in glioma cell lines. **A** qRT-PCR was used to determine the expression level of lncRNA NUTM2A-AS1 in glioma cell lines. **B** qRT-PCR was used to determine that the expression level of miR-376a-3p in glioma cell lines. **p < 0.01 vs. HEB group

To investigate the role of lncRNA NUTM2A-AS1 and miR-376a-3p in glioma, the expression levels of lncRNA NUTM2A-AS1 and miR-376a-3p in human glioma cell lines (U251, T98-G, A172) and glial cell lines (HEB) were measured by qRT-PCR. The results showed that lncRNA NUTM2A-AS1 expression was significantly higher in U251, T98-G and A172 cells compared to HEB cells, while miR-376a-3p expression was significantly downregulated (Fig. 2A, B). These results indicated that lncRNA NUTM2A-AS1 and miR-376a-3p are aberrantly expressed in glioma cell lines.

### LncRNA NUTM2A-AS1 negatively regulates miR-376a-3p expression in U251 cells

**Fig. 3:**
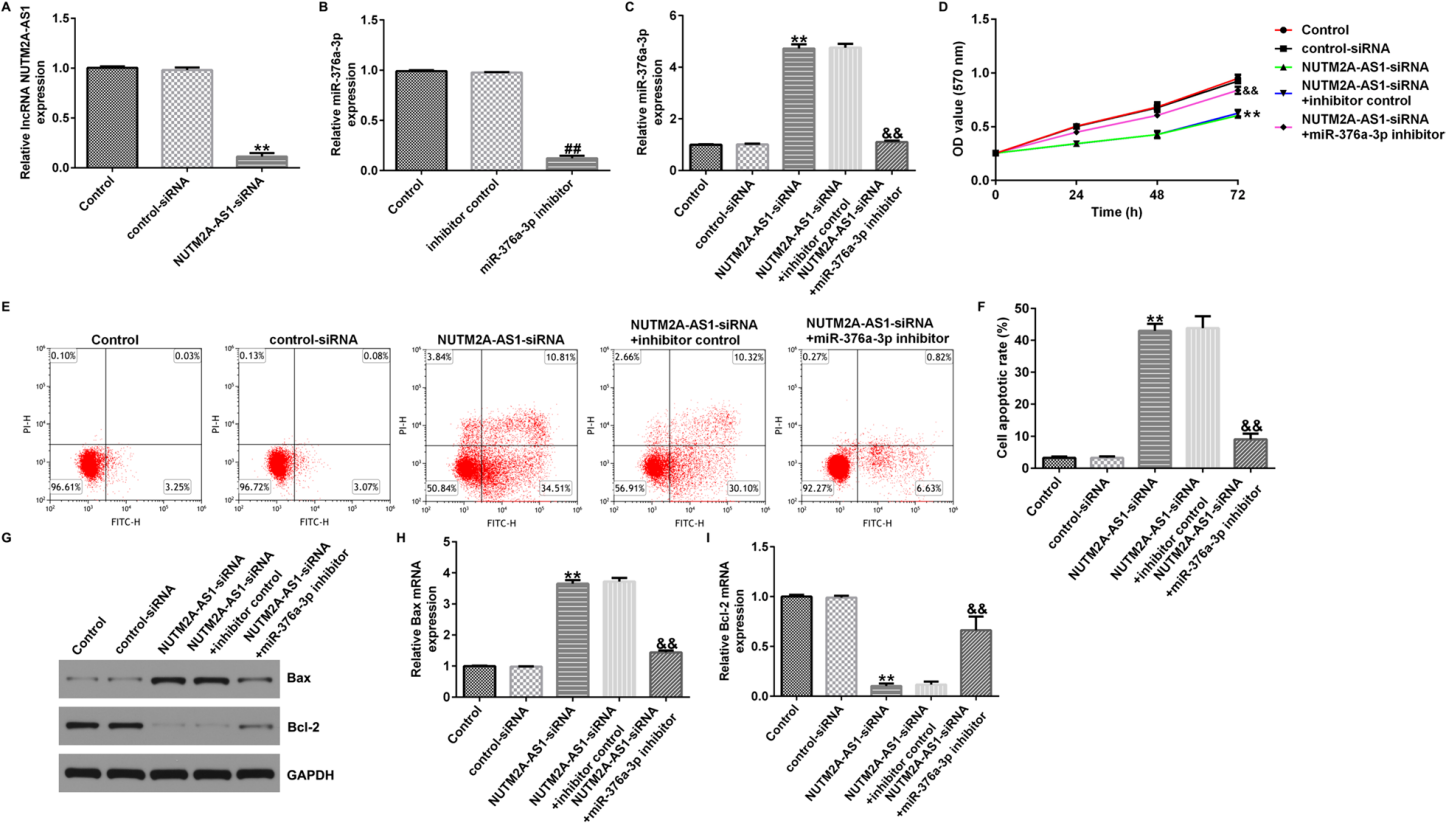
LncRNA NUTM2A-AS1 negatively regulates miR-376a-3p in U251 cell line. **A**–**C** qRT-PCR was performed to analyze the expression of lncRNA NUTM2A-AS1 and miR-376a-3p in U251 cells. **D** MTT assay was conducted to assess the cell viability of U251 cells. **E**, **F** Flow cytometry was used to quantify the apoptosis of U251 cells. **G** Western blot assay was conducted to analyze the protein expression of Bax and Bcl-2. **H** qRT-PCR was conducted to analyze the mRNA expression of Bax. **I** qRT-PCR was conducted to analyze the mRNA expression of Bcl-2. **p < 0.01 vs. control-siRNA; ^##^p < 0.01 vs. inhibitor control; ^&&^p < 0.01 vs. NUTM2A-AS1-siRNA + inhibitor control

To investigate the relationship between lncRNA NUTM2A-AS1 and miR-376a-3p in glioma, we transfected control-siRNA, lncRNA NUTM2A-AS1-siRNA, inhibitor control, miR-376a-3p inhibitor, lncRNA NUTM2A-AS1-siRNA + inhibitor control or lncRNA NUTM2A-AS1-siRNA + miR-376a-3p inhibitor into U251 and A172 cells. After transfection for 48 h, the transfection efficiency was detected by qRT-PCR. The results showed that lncRNA NUTM2A-AS1-siRNA significantly reduced the expression of lncRNA NUTM2A-AS1 in U251 cells (Fig. 3A). Compared with inhibitor control group, miR-376a-3p inhibitor significantly decreased the expression of miR-376a-3p in U251 cells (Fig. 3B). As shown in Fig. 3C, downregulation of lncRNA NUTM2A-AS1 enhanced the expression of miR-376a-3p, and this effect was reversed by miR-376a-3p inhibitor. Similar results were observed in A172 cells (Supplementary Fig. 1). These findings suggest that lncRNA NUTM2A-AS1 negatively regulates the expression of miR-376a-3p in glioma cells.

### Downregulation of lncRNA NUTM2A-AS1 affects proliferation and apoptosis of glioma cells through upregulation of miR-376a-3p

Next, we analyzed the effects of lncRNA NUTM2A-AS1 on the proliferation and apoptosis of U251 cells by loss-function experiments. The results of MTT assay showed that the viability of U251 cells was significantly reduced after silencing of lncRNA NUTM2A-AS1 (Fig. 3D), and this effect was reversed by miR-376a-3p inhibitor. Flow cytometry results showed that downregulation of lncRNA NUTM2A-AS1 significantly increased the apoptosis of U521 cells (Fig. 3E, F). In addition, western blotting and qRT-PCR results showed that lncRNA NUTM2A-AS1-siRNA transfected cells had increased protein and mRNA levels of Bax (Fig. 3G, H), while the protein and mRNA expression of Bcl-2 was decreased (Fig. 3G, I). These effects were significantly reversed after co-transfection with miR-376a-3p inhibitor, indicating that downregulation of lncRNA NUTM2A-AS1 and upregulation of miR-376a-3p could regulate proliferation and apoptosis in glioma cells.

### YAP1 is a direct target of miR-376a-3p

**Fig. 4:**
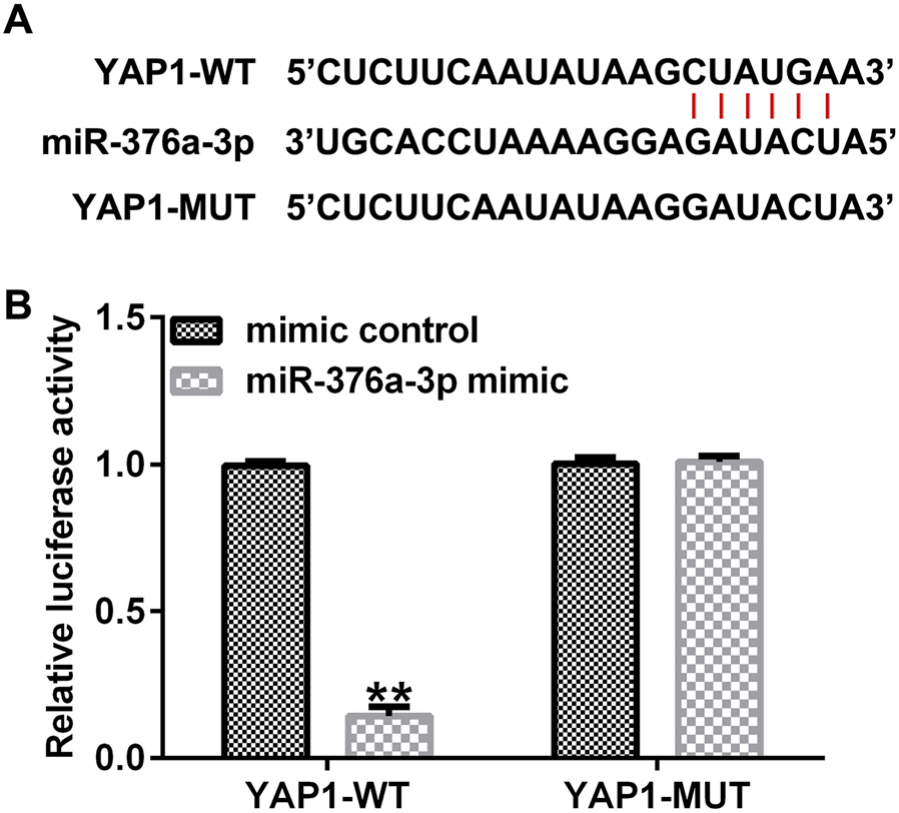
YAP1 is a direct target of miR-376a-3p. **A** YAP1 was predicted as a potential target of miR-376a-3p with StarBase. **B** Dual luciferase reporter assay was performed to confirm the interaction between miR-376a-3p and YAP1. **p < 0.01 vs. mimic control group

To further confirm the molecular regulation mechanism of miR-376a-3p in glioma, we analyzed the potential targets of miR-376a-3p through the StarBase database. The results showed that there is a direct binding site between YAP1 and miR-376a-3p (Fig. 4A). In addition, the results of the dual luciferase reporter showed that the relative luciferase activity of YAP1-WT was significantly reduced after co-transfection of miR-376a-3p mimic with YAP1-WT (Fig. 4B). These results confirm that YAP1 is a target gene for miR-376a-3p.

### Expression levels of YAP1 in glioma cell lines

**Fig. 5:**
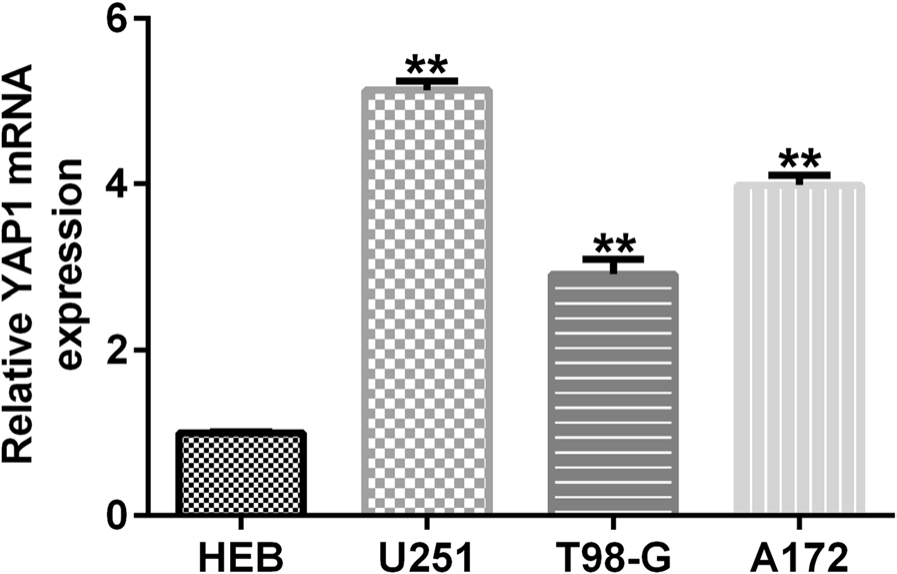
Expression levels of YAP1 in glioma cell lines. qRT-PCR was used to determine the expression level of YAP1 in glioma cell lines. **p < 0.01 vs. HEB group

Next, we examined the expression levels of YAP1 in glioma cell lines (U251, T98-G, A172) by qRT-PCR. The results showed that YAP1 expression was significantly higher in glioma cell lines incuding U251, T98-G and A172 cells than in HEB cell lines (Fig. 5A). These results suggest that YAP1 expression is increased in glioma cell lines.

### MiR-376a-3p negatively regulates YAP1 in human glioma cells

**Fig. 6:**
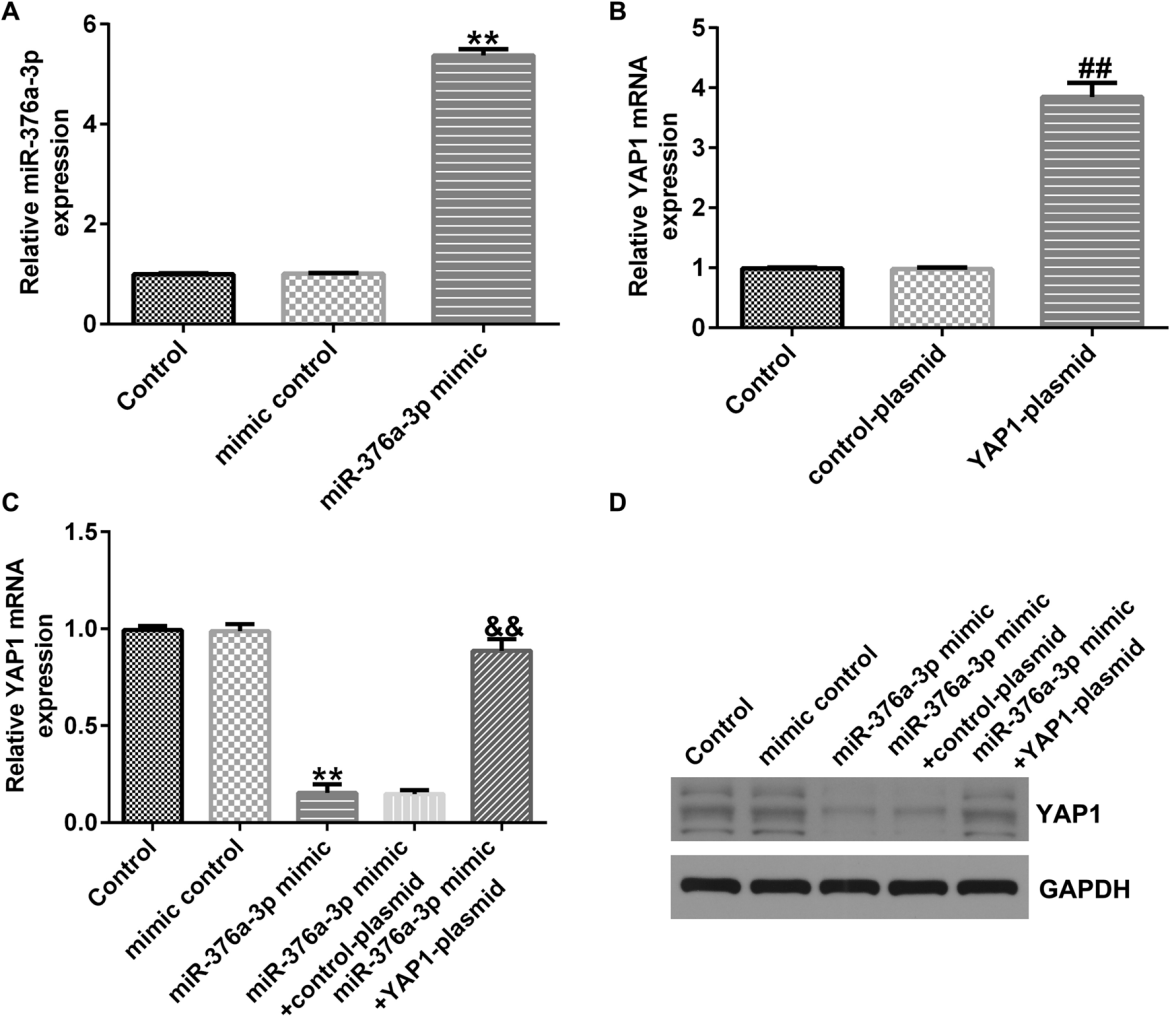
MiR-376a-3p negatively regulates YAP1 expression in U251 cell line. **A** qRT-PCR was performed to analyze the expression of miR-376a-3p in U251 cells. **B** qRT-PCR was performed to analyze the mRNA expression of YAP1 in U251 cells. **C**, **D** qRT-PCR and western blot assay were performed to analyze the mRNA and protein expression of YAP1. **p < 0.01 vs. mimic control; ^##^p < 0.01 vs. control-plasmid; ^&&^p < 0.01 vs. miR-376a-3p mimic + control-plasmid

U251 and A172 cells were transfected with mimic control, miR-376a-3p mimic, control-plasmid, YAP1-plasmid, miR-376a-3p mimic + control-plasmid, or miR-376a-3p mimic + YAP1-plasmid. After 48 h of transfection, we first examined the transfection efficiency by qRT-PCR. The miR-376a-3p mimic significantly increased the expression of miR-376a-3p in U251 cells compared to the mimic control group (Fig. 6A). YAP1-plasmid significantly increased the mRNA expression of YAP1 compared to control-plasmid group (Fig. 6B). In addition, YAP1 was significantly reduced in U251 cells after transfection with miR-376a-3p mimic. However, this effect was reversed after co-transfection with YAP1-plasmid (Fig. 6C, D). Similar results were observed in A172 cells (Supplementary Fig. 2).

### MiR-376a-3p affects proliferation and apoptosis of human glioma cells through downregulation of YAP1

**Fig. 7:**
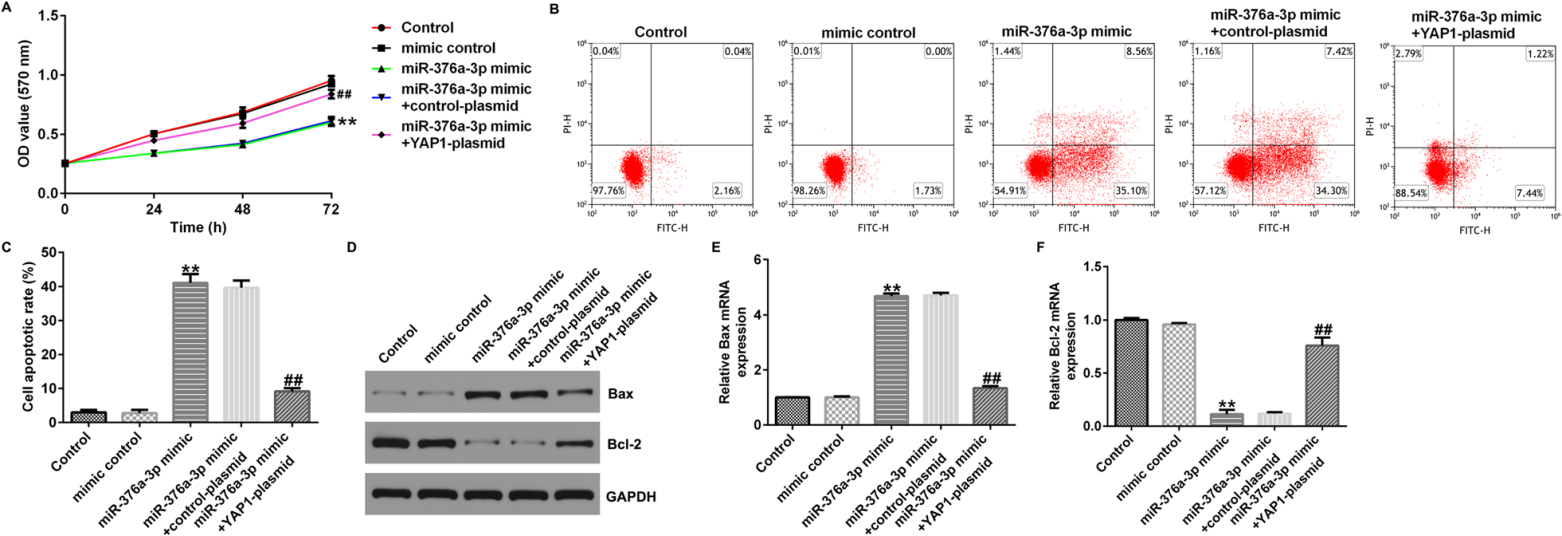
MiR-376a-3p affects proliferation and apoptosis of U251 cells through the downregulation of YAP1. **A** MTT assay was conducted to assess the cell viability of U251 cells. **B**, **C** Flow cytometry was used to quantify the apoptosis of U251 cells. **D** Western blot assay was conducted to analyze the protein expression of Bax and Bcl-2. **E** qRT-PCR was conducted to analyze the mRNA expression of Bax. **F** qRT-PCR was conducted to analyze the mRNA expression of Bcl-2. **p < 0.01 vs. mimic control; ^##^p < 0.01 vs. miR-376a-3p mimic + control-plasmid

Further studies revealed that miR-376a-3p affected the proliferation and apoptosis of U251 and A172 cells. MTT results showed that miR-376a-3p mimic significantly decreased the viability of U251 cells (Fig. 7A). Flow cytometry results showed that miR-376a-3p mimic significantly promoted apoptosis in U251 cells compared to the mimic control group (Fig. 7B, C). In addition, western blotting and qRT-PCR results showed that miR-376a-3p mimic increased the protein and mRNA expression of Bax (Fig. 7D, E), while the protein and mRNA expression of Bcl-2 were significantly reduced (Fig. 7D, F). However, all these effects were reversed by YAP1-plasmid. Moreover, our findings indicated that miR-376a-3p mimic inhibited cell proliferation, enhanced cell apoptosis, increased Bax expression, and decreased Bcl-2 expression in A172 cells were significantly eliminated by YAP1-plasmid (Supplementary Fig. 3). These data suggest that upregulation of miR-376a-3p can reduce cell proliferation and promote apoptosis via down-regulating YAP1 expression.

### YAP1 enhances proliferation and reduces apoptosis of human glioma cells

**Fig. 8:**
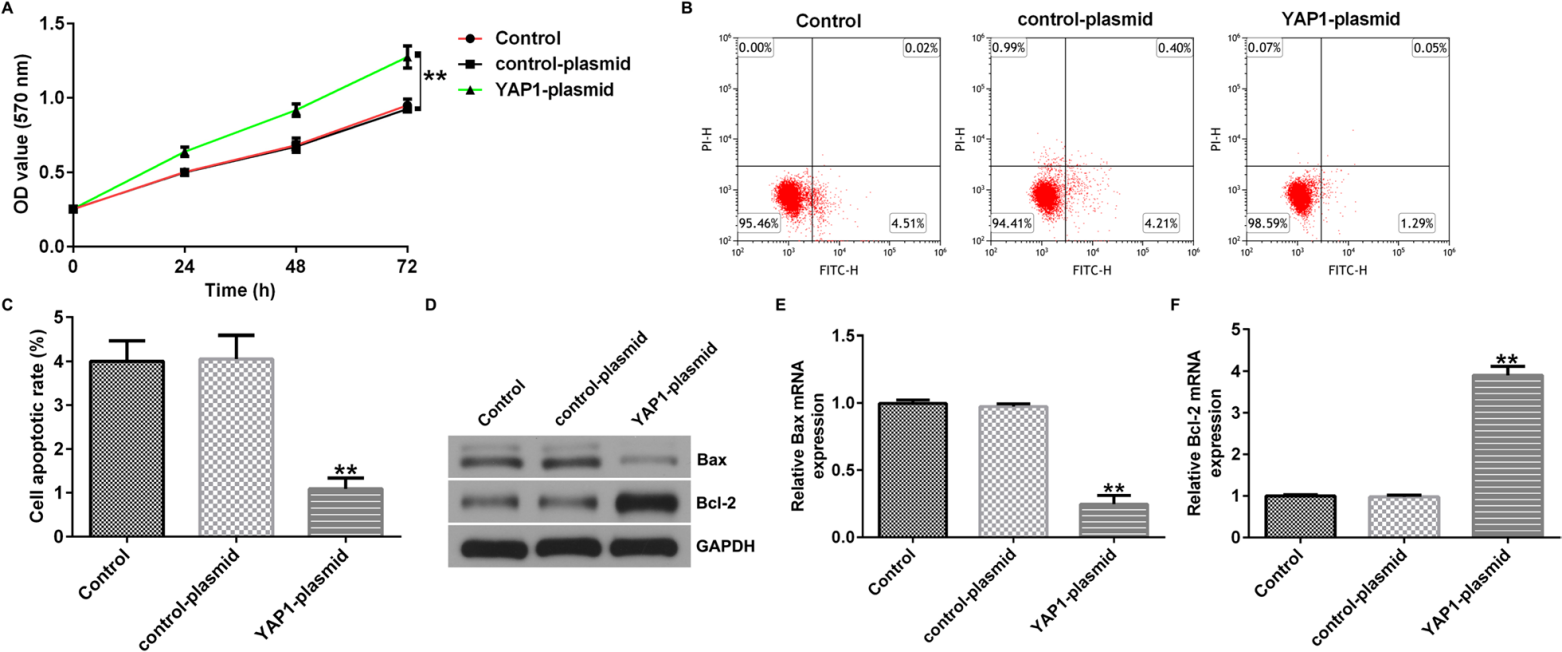
YAP1 enhances proliferation and reduces apoptosis of U251 cells. **A** MTT assay was conducted to assess the cell proliferation of U251 cells. **B**, **C** Flow cytometry was used to quantify the apoptosis of U251 cells. **D** Western blot assay was conducted to analyze the protein expression of Bax and Bcl-2. **E** qRT-PCR was conducted to analyze the mRNA expression of Bax. **F** qRT-PCR was conducted to analyze the mRNA expression of Bcl-2. **p < 0.01 vs. control-plasmid group

We then explored the role of YAP1 in U251 and A172 cells proliferation and apoptosis. The data indicated that compared with the control-plasmid group, YAP1-plasmid significantly enhanced the proliferation of U251 cells (Fig. 8A), reduced cell apoptosis (Fig. 8B, C), decreased Bax expression (Fig. 8D, E), and enhanced Bcl-2 expression (Fig. 8D, F). In A172 cells, YAP1-plasmid significantly promoted the cell proliferation, reduced cell apoptosis, decreased Bax expression, and enhanced Bcl-2 expression (Supplementary Fig. 4).

### LncRNA NUTM2A-AS1 positively regulates of YAP1 expression in human glioma cells

**Fig. 9:**
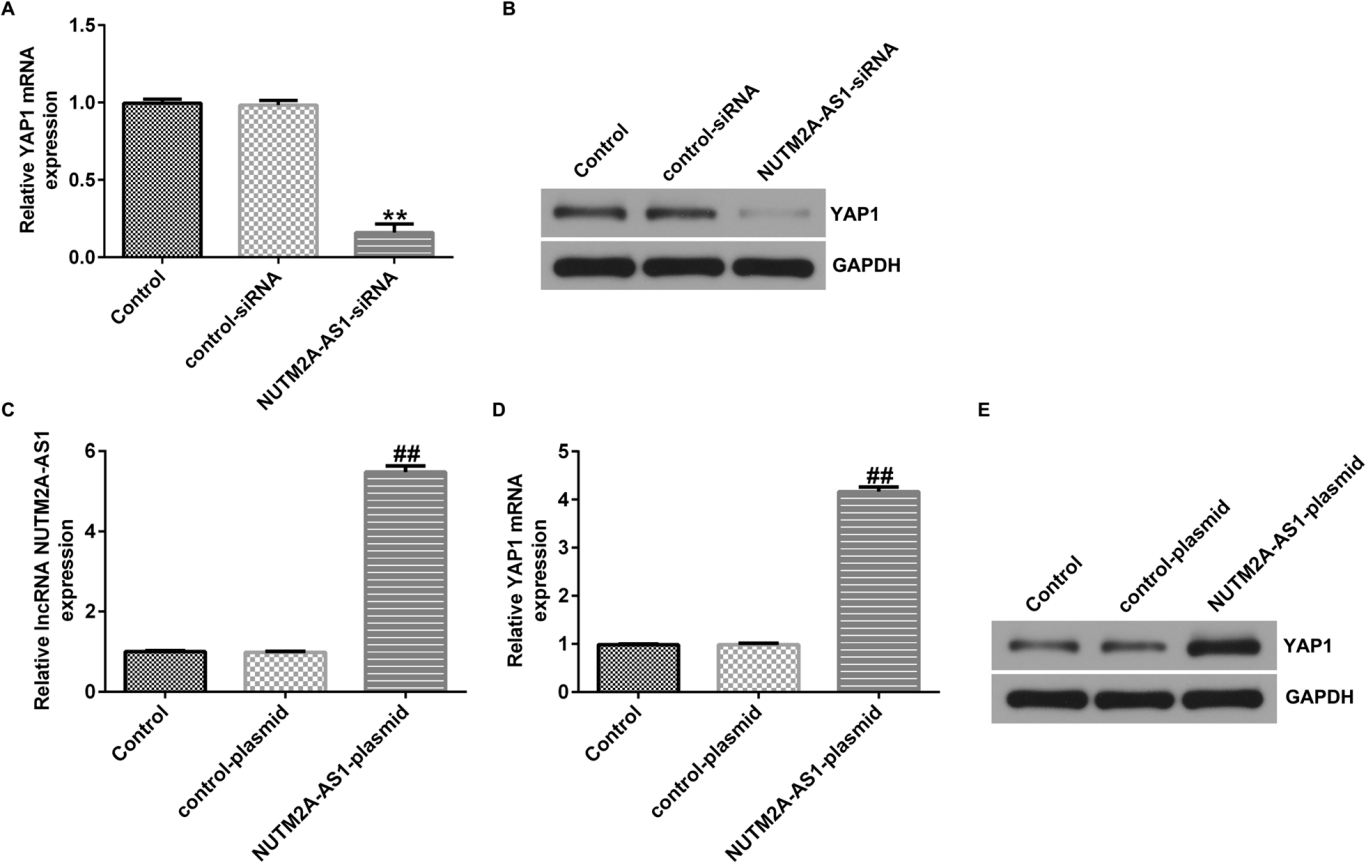
LncRNA NUTM2A-AS1 positively regulates of YAP1 expression in U251 cells. **A**, **B** The mRNA and protein level of YAP1 in U251 cells transfected with NUTM2A-AS1-siRNA or control-siRNA was determined using qRT-PCR and western blot assay. **C** The level of lncRNA NUTM2A-AS1 in U251 cells transfected with NUTM2A-AS1-plasmid or control-plasmid was determined using qRT-PCR. **D**, **E** The mRNA and protein level of YAP1 in U251 cells transfected with NUTM2A-AS1-plasmid or control-plasmid was determined using qRT-PCR and western blot assay. **p < 0.01 vs. control-siRNA group; ^##^p < 0.01 vs. control-plasmid group

Finally, we investigated the relationship between lncRNA NUTM2A AS1 and YAP1 in human glioma cells. The findings suggested that compared with the control-siRNA group, NUTM2A AS1-siRNA significantly reduced YAP1 expression in U251 cells (Fig. 9A, B). Compared to the control-plasmid group, NUTM2A AS1-plasmid significantly enhanced lncRNA NUTM2A AS1 expression, and increased YAP1 expression in U251 cells (Fig. 9C–E). Similar results were observed in A172 cells (Supplementary Fig. 5). The findings suggested that lncRNA NUTM2A-AS1 positively regulates of YAP1 expression in human glioma cells.

## Discussion

Glioma is the most common malignant tumour in the brain and is characterized by high recurrence, high mortality and poor prognosis [5, 32]. In-depth studies on the pathogenesis of glioma are beneficial for the development of new therapeutic approaches. There is growing evidences that dysregulation of lncRNA is closely associated with the development of glioma [2, 10]. In biological activity, lncRNAs act as signaling mediators involved in gene activity regulation, protein modification and post-transcriptional regulation [2, 7]. Furthermore, an important mechanism of lncRNAs is that they can act as competitive endogenous RNAs (ceRNAs) or miRNA sponges to regulate mRNA expression [13, 33]. For example, lncRNA H19 can act as a ceRNA through the miR-138/HIF-1α axis to promote the proliferation and invasion of glioma cells [34]. In addition, lncRNA HOTAIR acts as a ceRNA to regulate HER2 expression in gastric cancer via miR-331-3p [35]. Various lncRNAs were found to be aberrantly expressed in glioma, such as lncRNA PVT1, lncRNA BCYRN1 and lncRNA DGCR5 [36–38]. Recently, lncRNA NUTM2A-AS1 was found to be an oncogene and upregulated in non-small cell lung cancer [19]. However, the role and molecular mechanism of lncRNA NUTM2A-AS1 in glioma have not been reported.

An increasing number of studies have shown that the lncRNA-miRNA-mRNA regulatory network plays an important role in glioma [39]. Previous reports have demonstrated that lncRNA NUTM2A-AS1 binds directly to miR-376a in gastric cancer cells and acts as an oncogene [20]. In this study, we found that lncRNA NUTM2A-AS1 could directly bind to miR-376a-3p, and lncRNA NUTM2A-AS1 was negatively correlated with miR-376a-3p in glioma cells. Furthermore, silencing of lncRNA NUTM2A-AS1 could enhance the expression of miR-376a-3p, thereby inhibiting the proliferation of glioma cells and inducing apoptosis. Next, the target relationship between miR-376a-3p and YAP1 was verified by bioinformatic analysis and dual fluorescein mycobacterial reporter assay. Over-expression of miR-376a-3p significantly inhibited proliferation and induced apoptosis in glioma cells through down-regulating YAP1 expression. Also, we found that lncRNA NUTM2A-AS1 positively regulates of YAP1 expression in human glioma cells. Thus, lncRNA NUTM2A-AS1 may regulate the proliferation and apoptosis of glioma cells through the miR-376a-3p/YAP1 axis. This may be a novel mechanism for glioma development, suggesting lncRNA NUTM2A-AS1 as a new potential therapeutic target for glioma.

This study for the first time reveals the effects and potential mechanisms of lncRNA NUTM2A-AS1 in glioma. However, the study was mainly explored at the cellular level, and further research is needed to increase the reliability of the results. For example, the role and mechanism of lncRNA NUTM2A-AS1 can be verified in vivo by constructing an animal model of glioma. In addition, the relationship between lncRNA NUTM2A AS1 and YAP1 has not been fully verified. Previous studies have found that YAP1 can bind to miRNAs, such as miR-622, miR-27b-3p and miR-195-5p, which are involved in regulating the proliferation, apoptosis and migration of glioma cell lines [18, 40, 41]. These results suggest whether lncRNA NUTM2A AS1 can regulate YAP1 expression through other miRNAs in addition to miR-376a-3p, thereby affecting glioma cell proliferation and apoptosis needs further exploration.

However, there were some limitations of this study. Firstly, the experiments were all performed in vitro, and no in vivo investigations were performed. Besides, the clinical relevance of the miR-376a-3p/NUTM2A-AS1/YAP1 axis in glioma was not investigated. Additionally, the study only focused on one specific lncRNA (NUTM2A-AS1) and its relationship with one specific miRNA (miR-376a-3p) and gene (YAP1). The mechanisms of other lncRNAs and their interactions with other miRNAs and genes may also contribute to the development of glioma, but were not investigated in this study. Further research is needed to explore the broader landscape of lncRNA-miRNA-gene regulatory networks in glioma. We will perform these issues in the future.

In summary, lncRNA NUTM2A-AS1 down-regulation inhibits glioma cell proliferation and induces glioma cell apoptosis by regulating the miR-376a-3p/YAP1 axis. These results imply that lncRNA NUTM2A-AS1 and miR-376a-3p may be potential targets for glioma, providing a new strategy for the treatment of glioma.

## Conclusion

Downregulation of lncRNA NUTM2A-AS1 plays a protective role in glioma through inhibiting proliferation and inducing apoptosis in human glioma cells via the regulation of miR-376a-3p/YAP1 axis.

## Materials and methods

### Cell culture and transfection

Human glioma cell lines (U251, T98-G, A172) and glial cell lines (HEB) were obtained from ATCC. Cells were cultured in DMEM medium containing 10% fetal bovine serum (FBS) in a 37℃, 5% CO_2_ incubator. After 24 h (h) of incubation, control small interfering RNA (control-siRNA), lncRNA NUTM2A-AS1-siRNA, inhibitor control, miR-376a-3p inhibitor, mimic control, miR-376a-3p mimic, control-plasmid, and YAP1-plasmid were transfected or co-transfected into U251 cells using Lipofectamine^®^ 2000 reagent (Invitrogen, USA), according to the manufacturer's protocol. After 48 h of transfection, cells were collected and subsequent experiments were performed.

### Quantitative reverse transcription polymerase chain reaction (qRT-PCR)

Total RNA was extracted from cell samples using TRIzol reagent (Invitrogen) and reverse transcribed into cDNA using the PrimeScript RT kit (Takara) according to manufacturer's instructions. The cDNAs were then quantified with SYBR Premix reagent (Takara) on ABI 7500 system (Applied Biosystem). U6 and GAPDH were used as internal controls and the primer sequences were shown in Table 1.

**Table 1:** Primer sequences for PCR

Gene	Forward sequence (5′–3′)	Reverse sequence (5′–3′)
lncRNA NUTM2A-AS1	TACCTCTAGTTCTTCCCG GC	TTTTGCTTTTCTCCTGGCCC
miR-376a-3p	TGCACCTAAAAGGAG	GTGCAGGGTCCGAGGT
Bax	CCCGAGAGGTCTTTTTCCGAG	CCAGCCCATGATGGTTCTGAT
Bcl-2	GGTGGGGTCATGTGTGTGG	CGGTTCAGGTACTCAGTCATCC
YAP1	GCAGTTGGGAGCTGTTTCTC	GCCATGTTGTTGTCTGATCG
U6	CTCGCTTCGGCAGCACAT ATA	AAATATGGAACGCTTCACGA
GAPDH	TTTGGTATCGTGGAAGGA CTC	GTAGAGGCAGGGATGATG TTCT

### Western blot assay

Total protein was extracted from cells with RIPA lysate, and protein concentration was determined using a BCA kit (Beyotime). Equal samples were separated by SDS-PAGE on 12% gels. The proteins were then transferred to PVDF membranes (Millipore) and blocked with a blocking solution containing 5% skimmed milk for 1 h. After blocking, the protein-containing membranes were incubated with primary antibodies overnight at 4 °C, including anti-YAP1, anti-Bax, anti-Bcl-2, anti-GAPDH. The next day, the membranes were washed with TBS containing 1% Tween, followed by incubation with the corresponding secondary antibodies for 2 h at room temperature. Protein bands were visualized with ECL detection kit (Beyotime).

### MTT assay for cell proliferation

Seeding 3000 cells into each well of a 96-well plate and the cells were transfected as described above. After 48 h of transfection, DMEM medium containing MTT (1 μg/μl) was added to each well and incubated for 4 h in an incubator at 37 °C with 5% CO_2_. Absorbance was measured with a spectrophotometer at 570 nm.

### Apoptosis detection by flow cytometry

After 48 h of transfection, the Annexin V-FITC/PI apoptosis detection kit (Beyotime Institute of Biotechnology, China) was used for cell apoptosis detection. In brief, 5 μl Annexin V FITC and 10 μl PI were incubated with the cells for 15 min at 4 °C without light, and then the cells were determined by a FACSCalibur flow cytometer (BD Biosciences, USA), and the data were analyzed with Kaluza Analysis (version 2.1.1.20653; Beckman Coulter, Inc., USA).

### Bioinformatics and dual luciferase reporting assay

The StarBase database (http://starbase.sysu.edu.cn/) was used to predict the binding sites among lncRNA NUTM2A-AS1, miR-376a-3p and YAP1. For dual luciferase reporting assay, NUTM2A-AS1-WT, NUTM2A-AS1-MUT, YAP1-WT or YAP1-MUT were co-transfected into U251 cells with miR-376a-3p mimic or mimic control. After 48 h of transfection, cells were collected and luciferase activity was assessed using a dual luciferase assay kit (Solarbio).

### Statistical analysis

Data were statistically analyzed using SPSS 20.0 (IBM Corp., USA). The results were expressed in terms of mean ± standard deviation (SD). Analyses were performed using unpaired Student's t-tests or one-way ANOVA, and p < 0.05 indicated statistical significance.

## Supplementary Information

Supplementary Material 1: Figure 1. LncRNA NUTM2A-AS1 negatively regulates miR-376a-3p in A172 cell line. (A-C) qRT-PCR was performed to analyze the expression of lncRNA NUTM2A-AS1 and miR-376a-3p in A172 cells; (D) MTT assay was conducted to assess the cell viability of A172 cells; (E–F) Flow cytometry was used to quantify the apoptosis of A172 cells; (G) Western blot assay was conducted to analyze the protein expression of Bax and Bcl-2 in A172 cells; (H) qRT-PCR was conducted to analyze the mRNA expression of Bax in A172 cells; (I) qRT-PCR was conducted to analyze the mRNA expression of Bcl-2 in A172 cells. **p < 0.01 vs. control-siRNA; ##p < 0.01 vs. inhibitor control; &&p < 0.01 vs. NUTM2A-AS1-siRNA + inhibitor control.

Supplementary Material 2: Figure 2. MiR-376a-3p negatively regulates YAP1 expression in A172 cell line. (A) qRT-PCR was performed to analyze the expression of miR-376a-3p in A172 cells. (B) qRT-PCR was performed to analyze the mRNA expression of YAP1 in A172 cells. (C and D) qRT-PCR and western blot assay were performed to analyze the mRNA and protein expression of YAP1 in A172 cells. **p < 0.01 vs. mimic control; ##p < 0.01 vs. control-plasmid; &&p < 0.01 vs. miR-376a-3p mimic + control-plasmid.

Supplementary Material 3: Figure 3. MiR-376a-3p affects proliferation and apoptosis of A172 cells through the downregulation of YAP1. MTT assay was conducted to assess the cell viability of A172 cells; (B-C) Flow cytometry was used to quantify the apoptosis of A172 cells; (D) Western blot assay was conducted to analyze the protein expression of Bax and Bcl-2 in A172 cells; (E) qRT-PCR was conducted to analyze the mRNA expression of Bax in A172 cells; (F) qRT-PCR was conducted to analyze the mRNA expression of Bcl-2 in A172 cells. **p < 0.01 vs. mimic control; ##p < 0.01 vs. miR-376a-3p mimic + control-plasmid.

Supplementary Material 4: Figure 4. YAP1 enhances proliferation and reduces apoptosis of A172 cells. (A) MTT assay was conducted to assess the cell proliferation of A172 cells; (B-C) Flow cytometry was used to quantify the apoptosis of A172 cells; (D) Western blot assay was conducted to analyze the protein expression of Bax and Bcl-2 in A172 cells; (E) qRT-PCR was conducted to analyze the mRNA expression of Bax in A172 cells; (F) qRT-PCR was conducted to analyze the mRNA expression of Bcl-2 in A172 cells. **p < 0.01 vs. control-plasmid group.

Supplementary Material 5: Figure 5. LncRNA NUTM2A-AS1 positively regulates of YAP1 expression in A172 cells. (A and B) The mRNA and protein level of YAP1 in A172 cells transfected with NUTM2A-AS1-siRNA or control-siRNA was determined using qRT-PCR and western blot assay. (C) The level of lncRNA NUTM2A-AS1 in A172 cells transfected with NUTM2A-AS1-plasmid or control-plasmid was determined using qRT-PCR. (D and E) The mRNA and protein level of YAP1 in A172 cells transfected with NUTM2A-AS1-plasmid or control-plasmid was determined using qRT-PCR and western blot assay. **p < 0.01 vs. control-siRNA group; ##p < 0.01 vs. control-plasmid group.

## Data Availability

The datasets used and/or analyzed during the current study are available from the corresponding author on reasonable request.
