# Correction: LncRNA NUTM2A-AS1 silencing inhibits glioma via miR-376a-3p/YAP1 axis

**DOI:** 10.1186/s13008-025-00157-x

**Published:** 2025-08-22

**Authors:** Yuecheng Zeng, Zhenyu Yang, Yang Yang, Peng Wang

**Affiliations:** Department of Neurosurgery, Xiangyang Central Hospital, Affiliated Hospital of Hubei University of Arts and Science, No. 136 Jingzhou Street, Xiangcheng District, Xiangyang, 441021 China

**Correction to: Cell Division (2024) 19:17**

10.1186/s13008-024-00122-0

In this article [1], the author would like to correct the errors in Figs. 3, 7 and 8 and Supplement Figs. 1, 3 and 4. For completeness and transparency, the old incorrect and correct versions are displayed below.

Incorrect Figures:

**Fig. 3:**
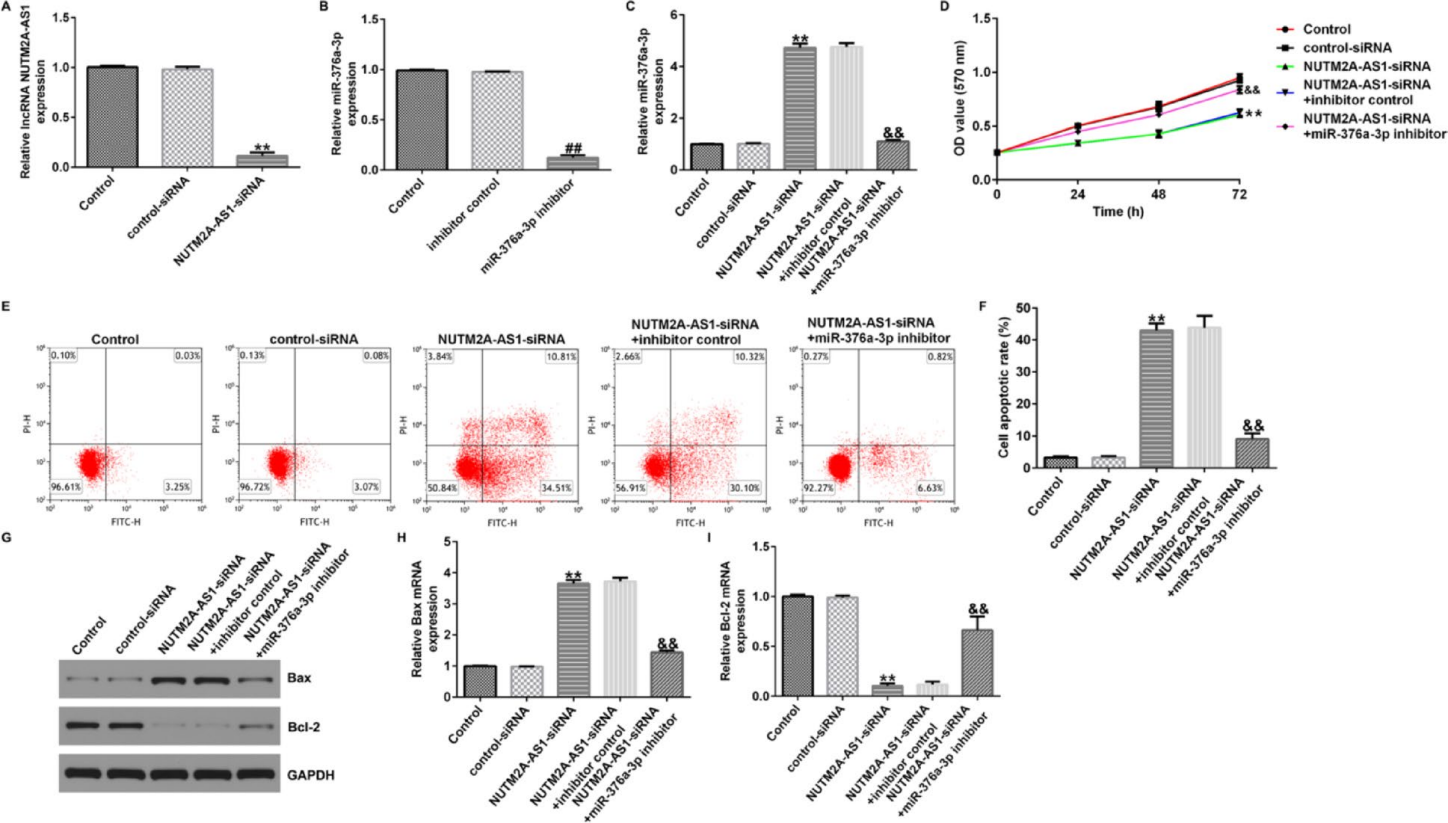
LncRNA NUTM2A-AS1 negatively regulates miR-376a-3p in U251 cell line. **A**–**C** qRT-PCR was performed to analyze the expression of lncRNA NUTM2A-AS1 and miR-376a-3p in U251 cells. **D** MTT assay was conducted to assess the cell viability of U251 cells. **E**, **F** Flow cytometry was used to quantify the apoptosis of U251 cells. G Western blot assay was conducted to analyze the protein expression of Bax and Bcl-2. H qRT-PCR was conducted to analyze the mRNA expression of Bax. I qRT-PCR was conducted to analyze the mRNA expression of Bcl-2. **p < 0.01 vs. control-siRNA; ^##^*p* < 0.01 vs. inhibitor control; ^&&^*p* < 0.01 vs. NUTM2A-AS1-siRNA + inhibitor control

**Fig. 7:**
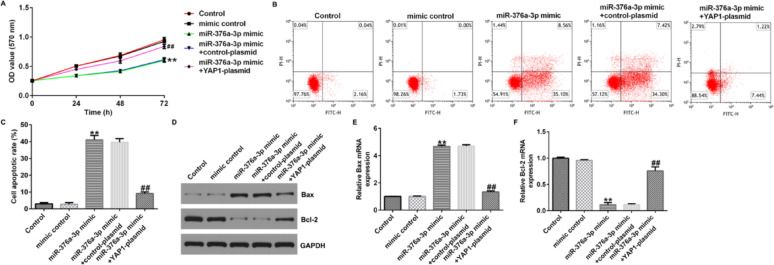
MiR-376a-3p affects proliferation and apoptosis of U251 cells through the downregulation of YAP1. **A** MTT assay was conducted to assess the cell viability of U251 cells. **B**, **C** Flow cytometry was used to quantify the apoptosis of U251 cells. **D** Western blot assay was conducted to analyse the protein expression of Bax and Bcl-2. **E** qRT-PCR was conducted to analyze the mRNA expression of Bax. **F** qRT-PCR was conducted to analyse the mRNA expression of Bcl-2. **p < 0.01 vs. mimic control; ^##^*p* < 0.01 vs. miR-376a-3p mimic + control-plasmid

**Fig. 8:**
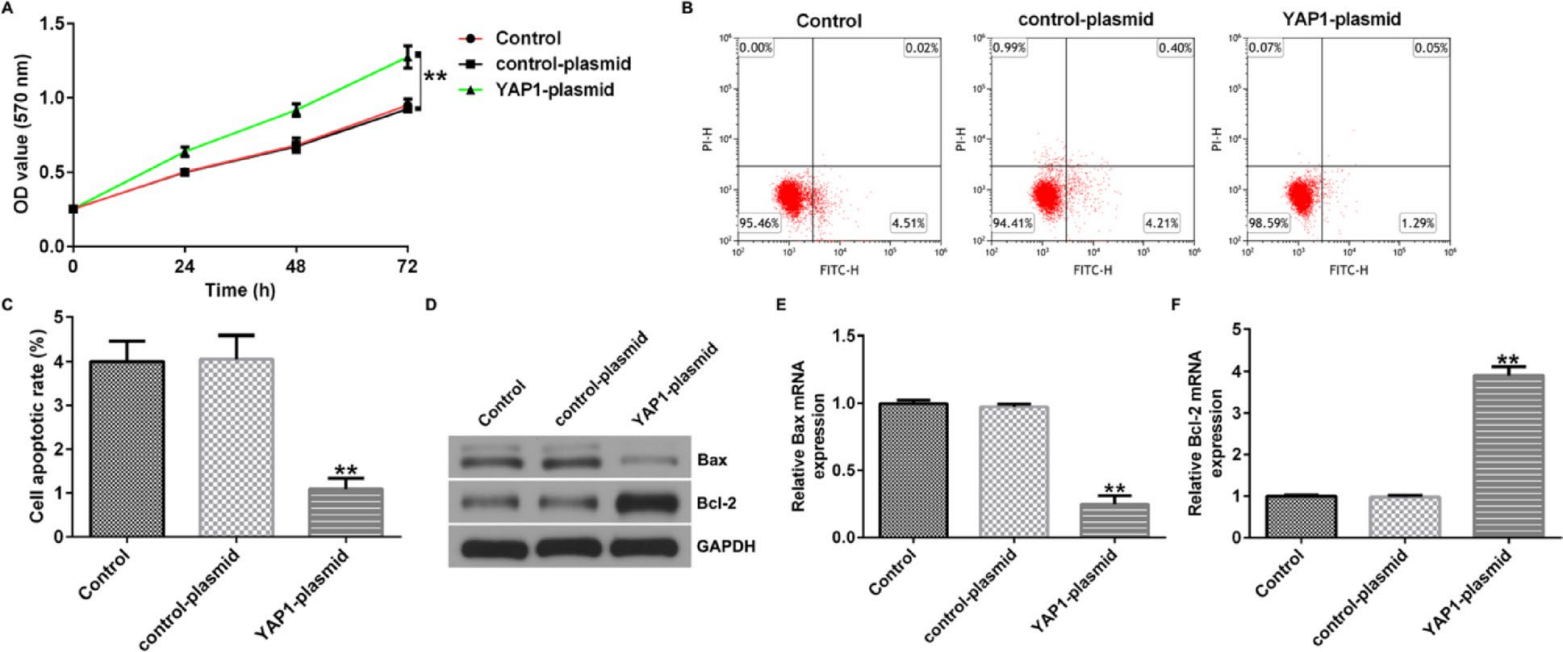
YAP1 enhances proliferation and reduces apoptosis of U251 cells. **A** MTT assay was conducted to assess the cell proliferation of U251 cells. **B**, **C** Flow cytometry was used to quantify the apoptosis of U251 cells. **D** Western blot assay was conducted to analyze the protein expression of Bax and Bcl-2. **E** qRT-PCR was conducted to analyze the mRNA expression of Bax. **F** qRT-PCR was conducted to analyze the mRNA expression of Bcl-2. **p < 0.01 vs. control-plasmid group

Correct Figures:

**Fig. 3:**
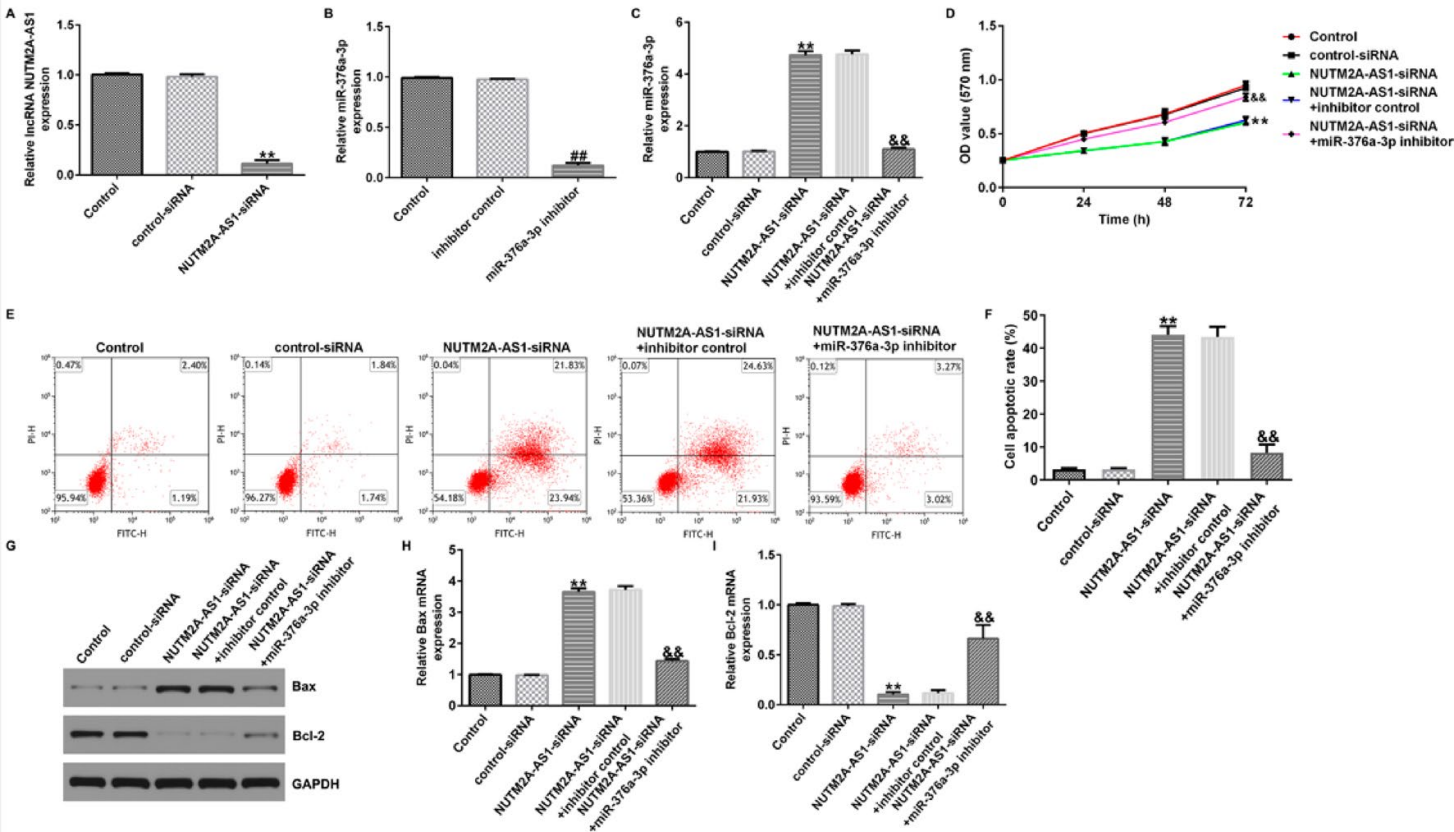
LncRNA NUTM2A-AS1 negatively regulates miR-376a-3p in U251 cell line. **A**–**C** qRT-PCR was performed to analyze the expression of lncRNA NUTM2A-AS1 and miR-376a-3p in U251 cells. **D** MTT assay was conducted to assess the cell viability of U251 cells. **E**, **F** Flow cytometry was used to quantify the apoptosis of U251 cells. G Western blot assay was conducted to analyze the protein expression of Bax and Bcl-2. H qRT-PCR was conducted to analyze the mRNA expression of Bax. I qRT-PCR was conducted to analyze the mRNA expression of Bcl-2. **p < 0.01 vs. control-siRNA; ^##^*p* < 0.01 vs. inhibitor control; ^&&^*p* < 0.01 vs. NUTM2A-AS1-siRNA + inhibitor control

**Fig. 7:**
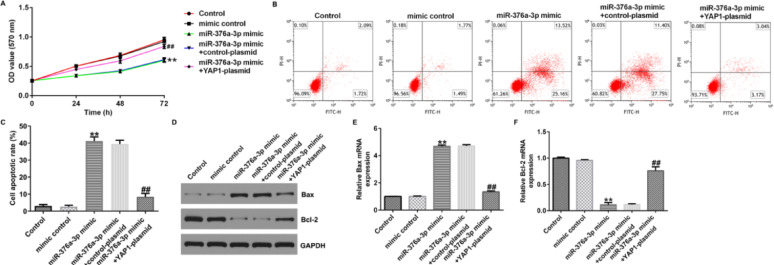
MiR-376a-3p affects proliferation and apoptosis of U251 cells through the downregulation of YAP1. **A** MTT assay was conducted to assess the cell viability of U251 cells. **B**, **C** Flow cytometry was used to quantify the apoptosis of U251 cells. **D** Western blot assay was conducted to analyse the protein expression of Bax and Bcl-2. **E** qRT-PCR was conducted to analyze the mRNA expression of Bax. **F** qRT-PCR was conducted to analyse the mRNA expression of Bcl-2. **p < 0.01 vs. mimic control; ^##^*p* < 0.01 vs. miR-376a-3p mimic + control-plasmid

**Fig. 8:**
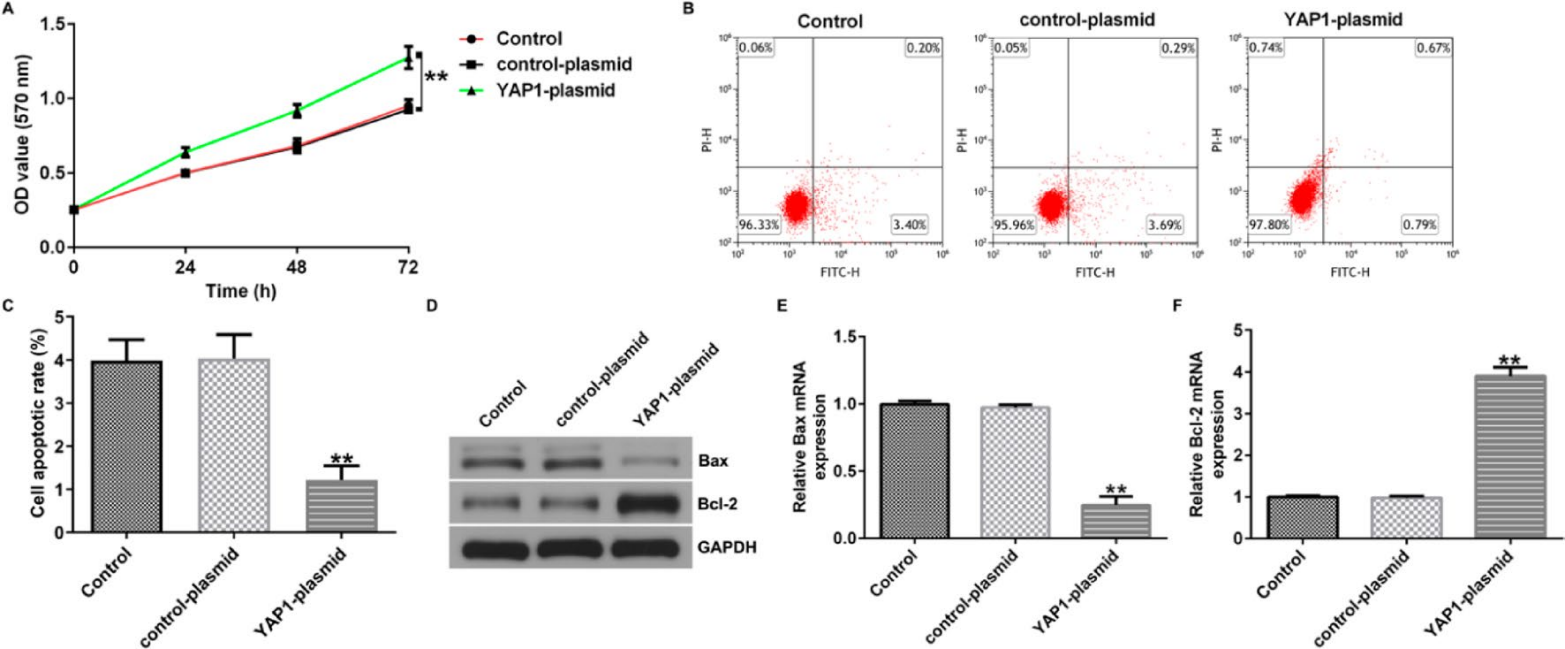
YAP1 enhances proliferation and reduces apoptosis of U251 cells. **A** MTT assay was conducted to assess the cell proliferation of U251 cells. **B**, **C** Flow cytometry was used to quantify the apoptosis of U251 cells. **D** Western blot assay was conducted to analyze the protein expression of Bax and Bcl-2. **E** qRT-PCR was conducted to analyze the mRNA expression of Bax. **F** qRT-PCR was conducted to analyze the mRNA expression of Bcl-2. **p < 0.01 vs. control-plasmid group

## Electronic supplementary material

Below is the link to the electronic supplementary material

Supplementary Material 1

Supplementary Material 2

Supplementary Material 3
