# Supplementary figures and images for: LncRNA NUTM2A-AS1 silencing inhibits glioma via miR-376a-3p/YAP1 axis
Source: Cell Div. 2024 May 10;19:17. doi: 10.1186/s13008-024-00122-0 (PMC11088135; doi:10.1186/s13008-024-00122-0)

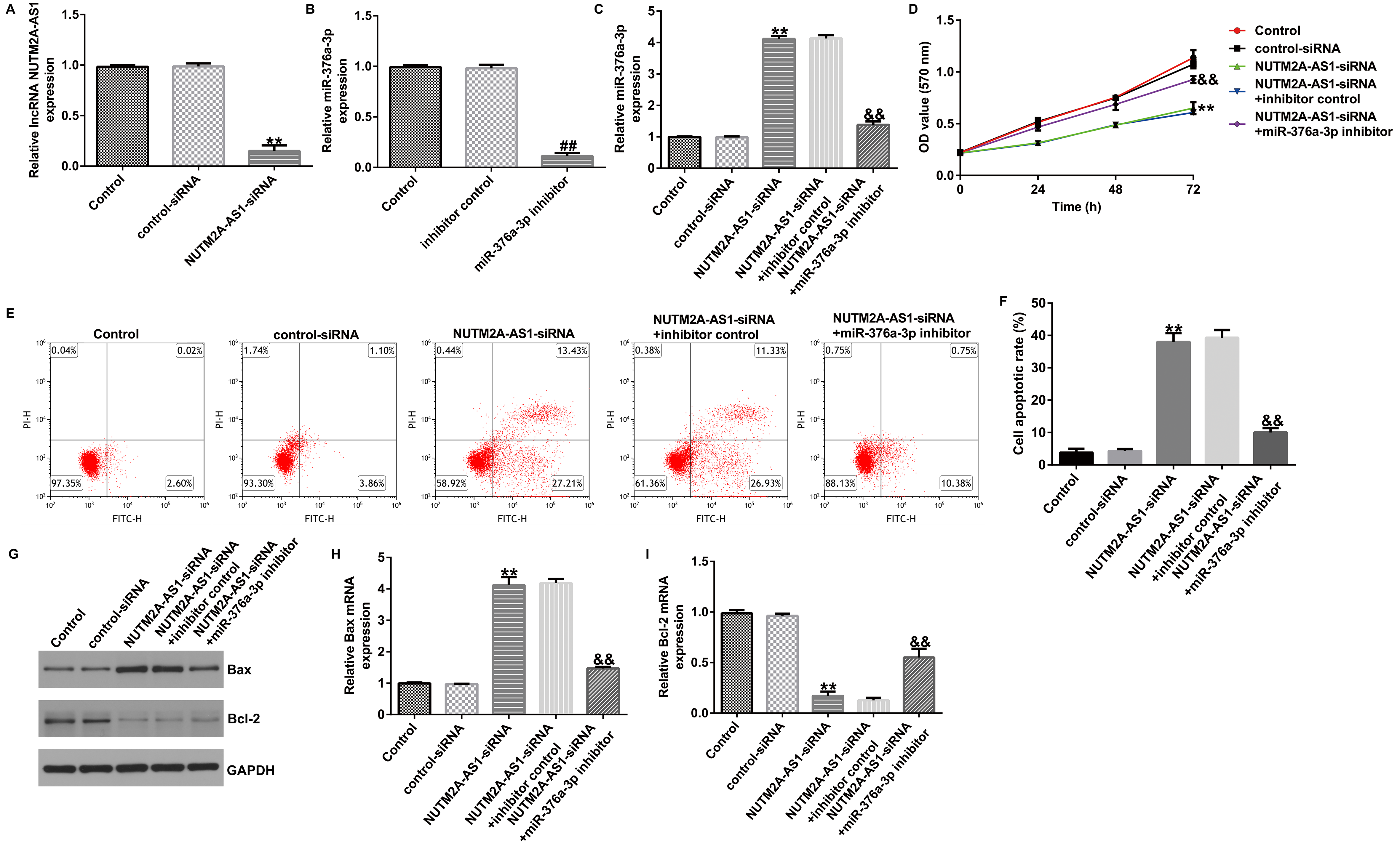

Supplement: Supplementary file 1 — Supplementary Material 1: Figure 1. LncRNA NUTM2A-AS1 negatively regulates miR-376a-3p in A172 cell line. (A-C) qRT-PCR was performed to analyze the expression of lncRNA NUTM2A-AS1 and miR-376a-3p in A172 cells; (D) MTT assay was conducted to assess the cell viability of A172 cells; (E–F) Flow cytometry was used to quantify the apoptosis of A172 cells; (G) Western blot assay was conducted to analyze the protein expression of Bax and Bcl-2 in A172 cells; (H) qRT-PCR was conducted to analyze the mRNA expression of Bax in A172 cells; (I) qRT-PCR was conducted to analyze the mRNA expression of Bcl-2 in A172 cells. **p < 0.01 vs. control-siRNA; ##p < 0.01 vs. inhibitor control; &&p < 0.01 vs. NUTM2A-AS1-siRNA + inhibitor control. [file 13008_2024_122_MOESM1_ESM.tif]

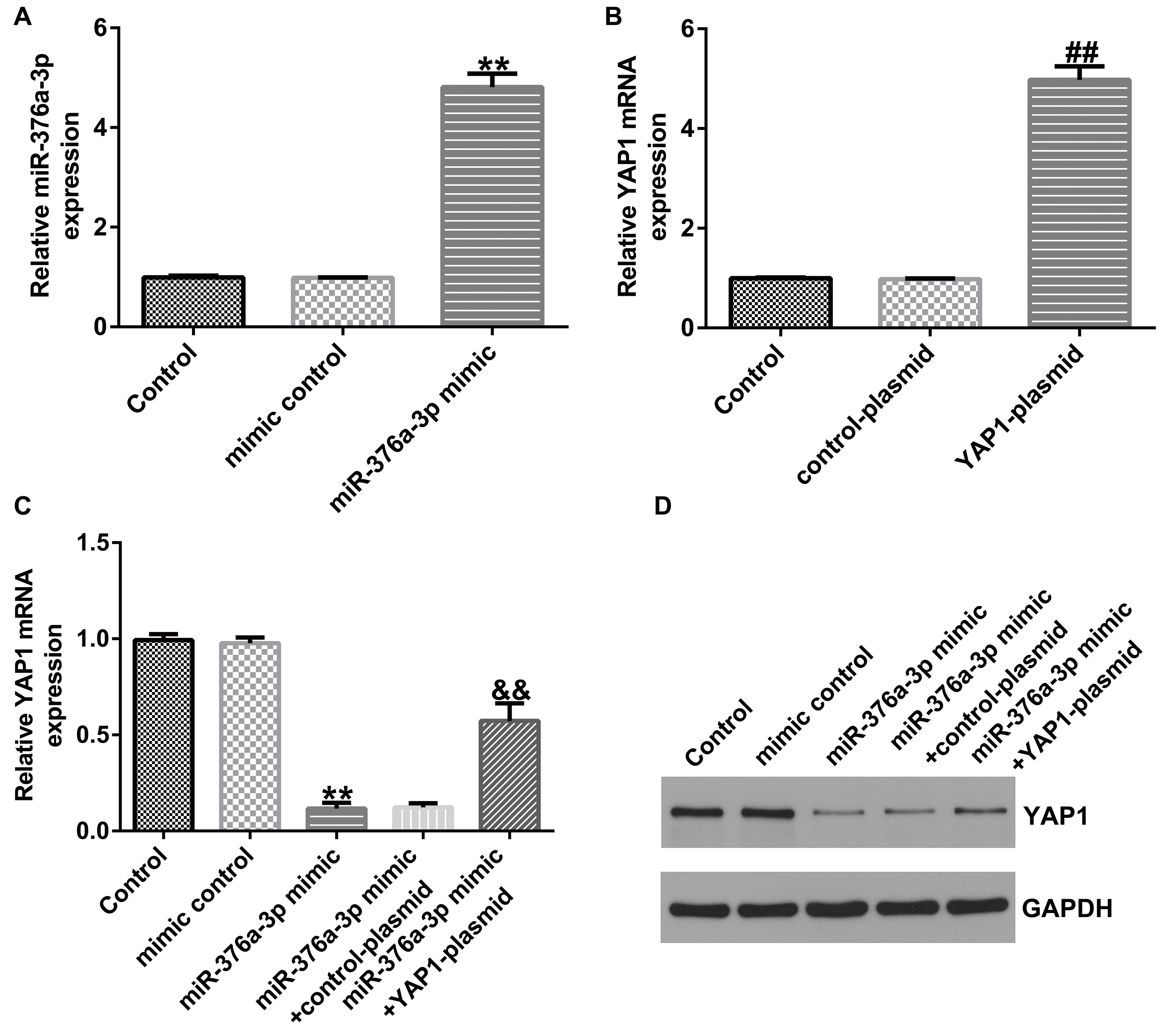

Supplement: Supplementary file 2 — Supplementary Material 2: Figure 2. MiR-376a-3p negatively regulates YAP1 expression in A172 cell line. (A) qRT-PCR was performed to analyze the expression of miR-376a-3p in A172 cells. (B) qRT-PCR was performed to analyze the mRNA expression of YAP1 in A172 cells. (C and D) qRT-PCR and western blot assay were performed to analyze the mRNA and protein expression of YAP1 in A172 cells. **p < 0.01 vs. mimic control; ##p < 0.01 vs. control-plasmid; &&p < 0.01 vs. miR-376a-3p mimic + control-plasmid. [file 13008_2024_122_MOESM2_ESM.tif]

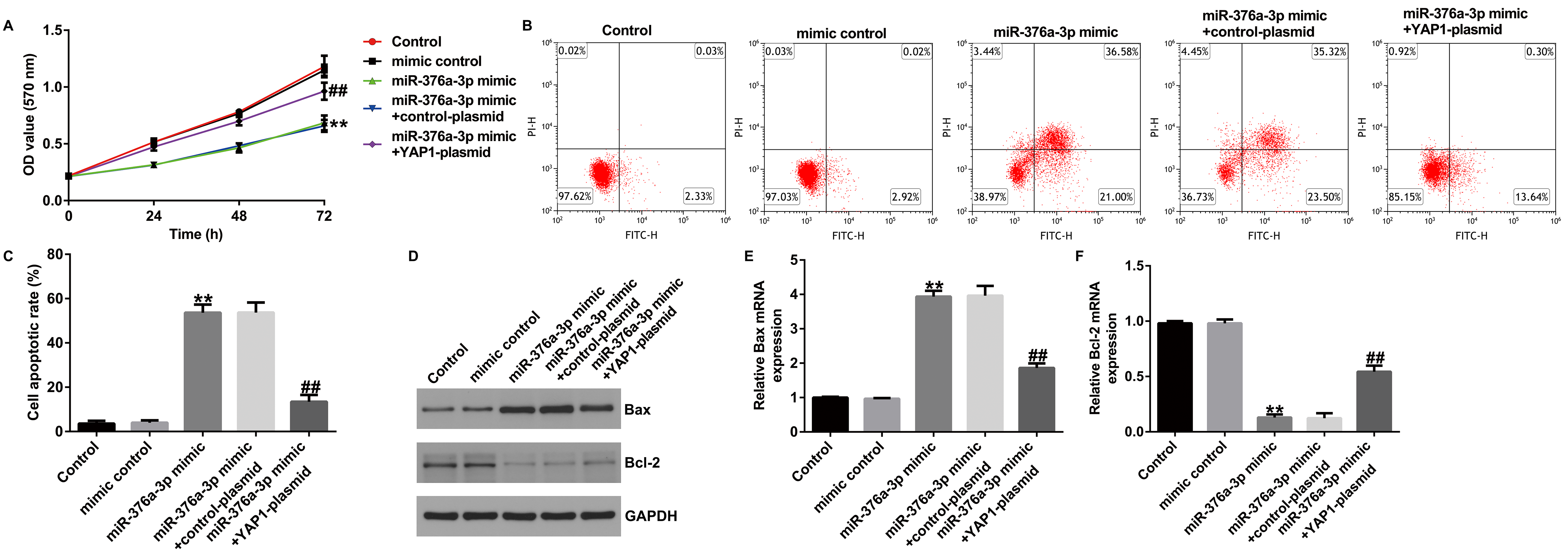

Supplement: Supplementary file 3 — Supplementary Material 3: Figure 3. MiR-376a-3p affects proliferation and apoptosis of A172 cells through the downregulation of YAP1. MTT assay was conducted to assess the cell viability of A172 cells; (B-C) Flow cytometry was used to quantify the apoptosis of A172 cells; (D) Western blot assay was conducted to analyze the protein expression of Bax and Bcl-2 in A172 cells; (E) qRT-PCR was conducted to analyze the mRNA expression of Bax in A172 cells; (F) qRT-PCR was conducted to analyze the mRNA expression of Bcl-2 in A172 cells. **p < 0.01 vs. mimic control; ##p < 0.01 vs. miR-376a-3p mimic + control-plasmid. [file 13008_2024_122_MOESM3_ESM.tif]

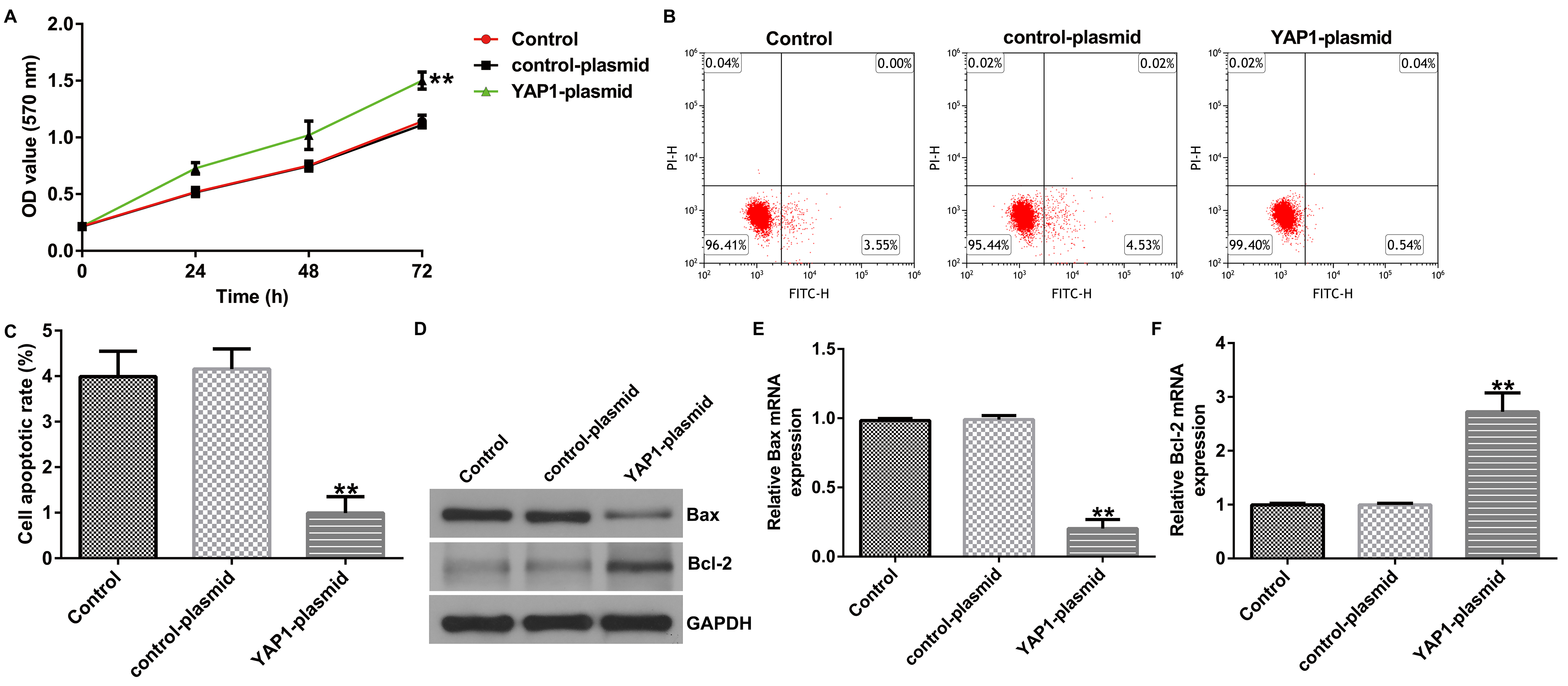

Supplement: Supplementary file 4 — Supplementary Material 4: Figure 4. YAP1 enhances proliferation and reduces apoptosis of A172 cells. (A) MTT assay was conducted to assess the cell proliferation of A172 cells; (B-C) Flow cytometry was used to quantify the apoptosis of A172 cells; (D) Western blot assay was conducted to analyze the protein expression of Bax and Bcl-2 in A172 cells; (E) qRT-PCR was conducted to analyze the mRNA expression of Bax in A172 cells; (F) qRT-PCR was conducted to analyze the mRNA expression of Bcl-2 in A172 cells. **p < 0.01 vs. control-plasmid group. [file 13008_2024_122_MOESM4_ESM.tif]

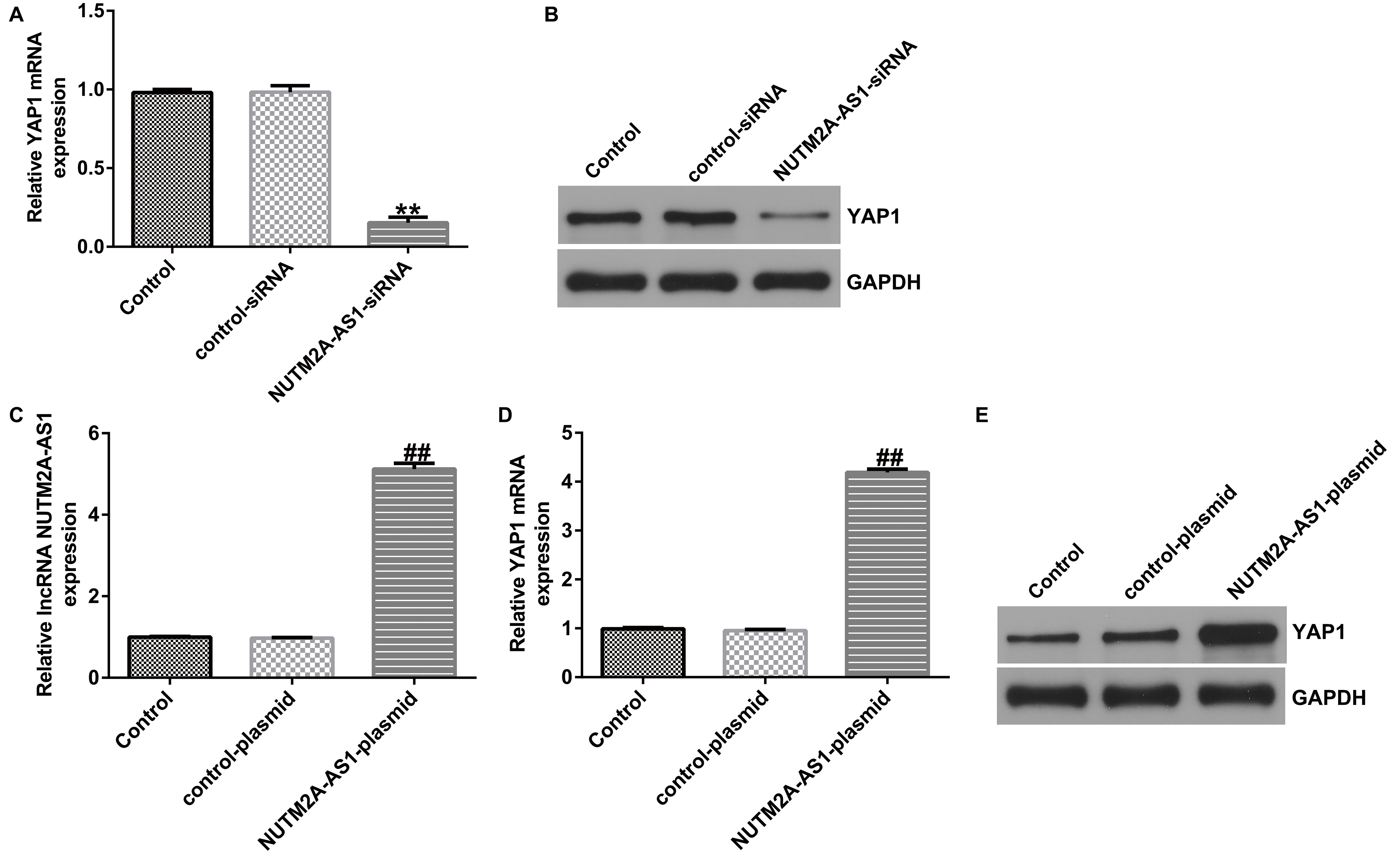

Supplement: Supplementary file 5 — Supplementary Material 5: Figure 5. LncRNA NUTM2A-AS1 positively regulates of YAP1 expression in A172 cells. (A and B) The mRNA and protein level of YAP1 in A172 cells transfected with NUTM2A-AS1-siRNA or control-siRNA was determined using qRT-PCR and western blot assay. (C) The level of lncRNA NUTM2A-AS1 in A172 cells transfected with NUTM2A-AS1-plasmid or control-plasmid was determined using qRT-PCR. (D and E) The mRNA and protein level of YAP1 in A172 cells transfected with NUTM2A-AS1-plasmid or control-plasmid was determined using qRT-PCR and western blot assay. **p < 0.01 vs. control-siRNA group; ##p < 0.01 vs. control-plasmid group. [file 13008_2024_122_MOESM5_ESM.tif]
