# Supplementary figures and images for: Correction: LncRNA NUTM2A-AS1 silencing inhibits glioma via miR-376a-3p/YAP1 axis
Source: Cell Div. 2025 Aug 22;20:21. doi: 10.1186/s13008-025-00157-x (PMC12374292; doi:10.1186/s13008-025-00157-x)

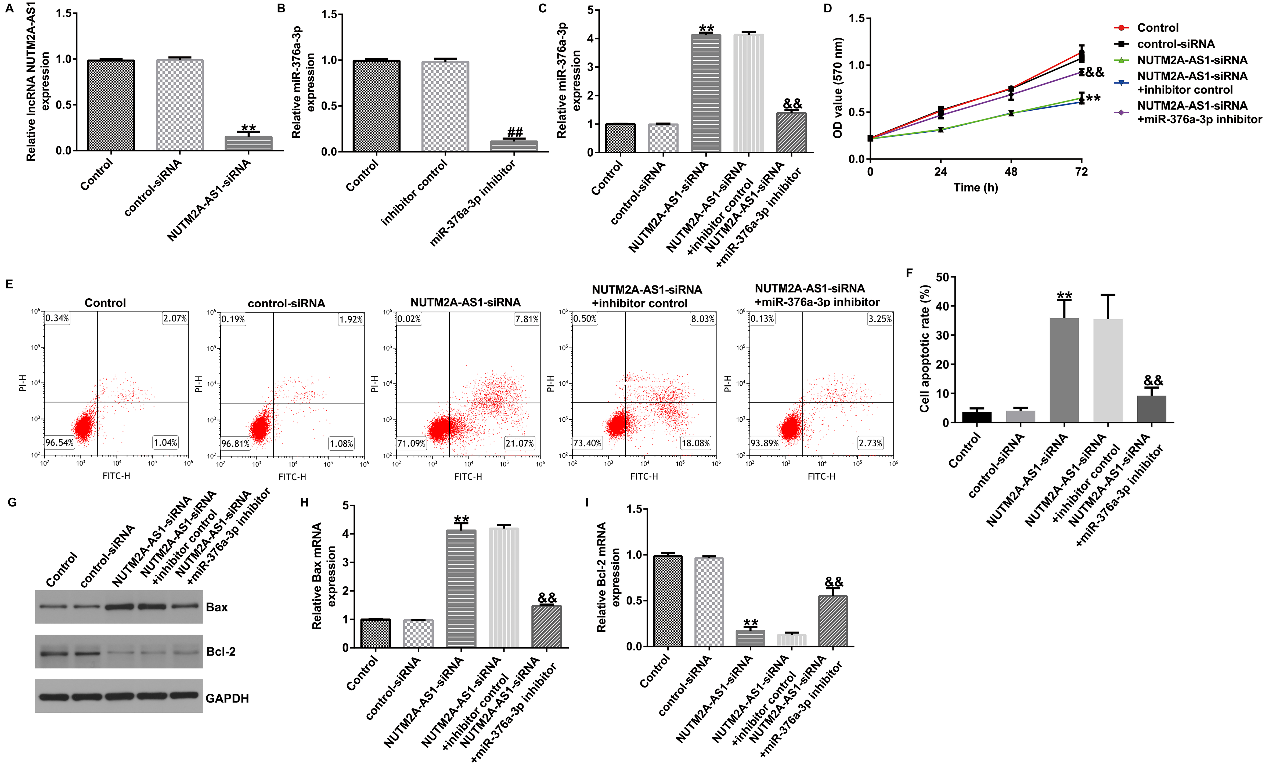


**Revised Supplementary Figure 1**

Supplement: Supplementary file 1 — Supplementary Material 1 [file 13008_2025_157_MOESM1_ESM.docx]

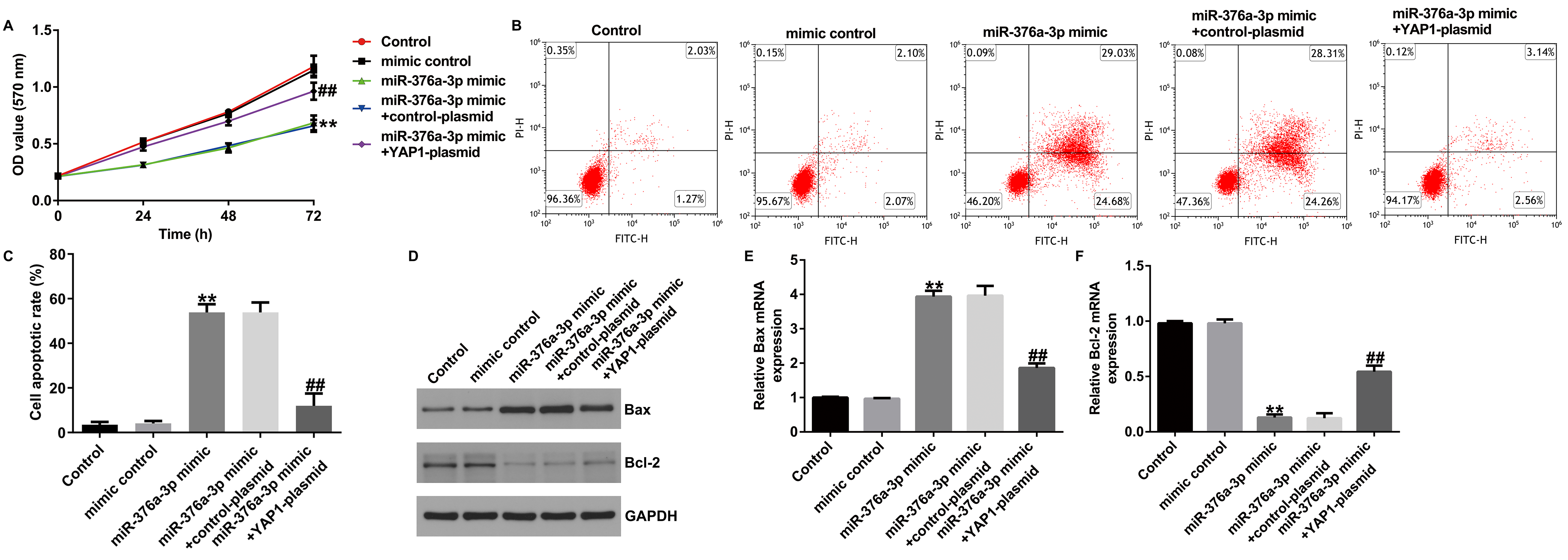

Supplement: Supplementary file 2 — Supplementary Material 2 [file 13008_2025_157_MOESM2_ESM.tif]

**
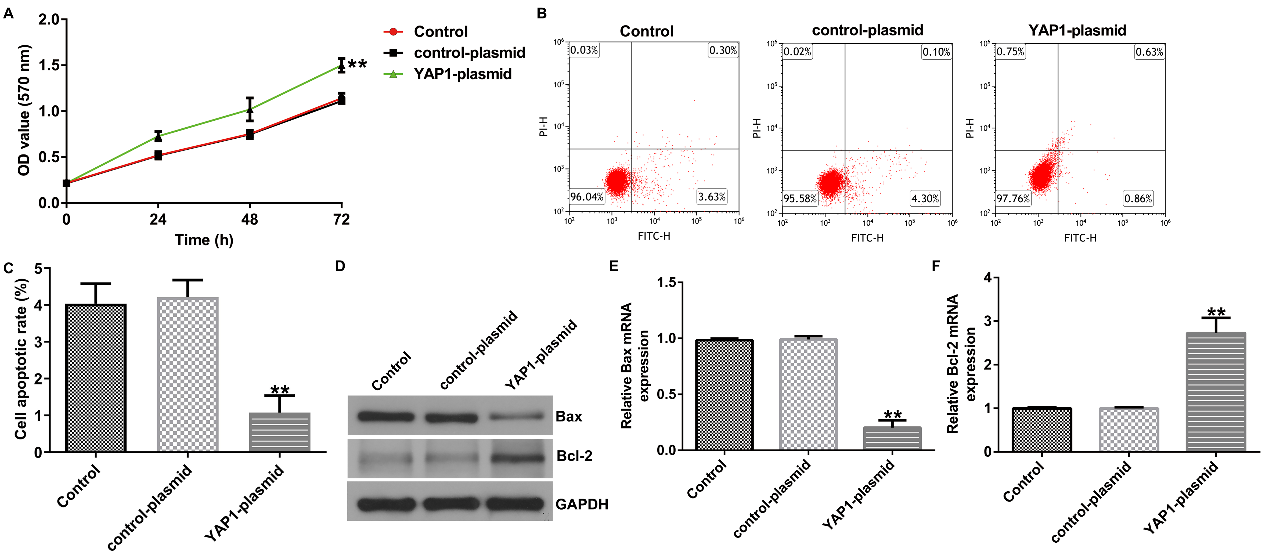
**

**Revised Supplementary Figure 4**

Supplement: Supplementary file 3 — Supplementary Material 3 [file 13008_2025_157_MOESM3_ESM.docx]
